# CRELD2, endoplasmic reticulum stress, and human diseases

**DOI:** 10.3389/fendo.2023.1117414

**Published:** 2023-03-02

**Authors:** Qin Tang, Qinhui Liu, Yanping Li, Li Mo, Jinhan He

**Affiliations:** ^1^ Department of Pharmacy, Institute of Metabolic Diseases and Pharmacotherapy, West China Hospital, Sichuan University, Chengdu, Sichuan, China; ^2^ Center of Gerontology and Geriatrics, National Clinical Research Center for Geriatrics, West China Hospital, Sichuan University, Chengdu, China

**Keywords:** CRELD2, endoplasmic reticulum stress, unfolded protein response, metabolism, human diseases

## Abstract

CRELD2, a member of the cysteine-rich epidermal growth factor-like domain (CRELD) protein family, is both an endoplasmic reticulum (ER)-resident protein and a secretory factor. The expression and secretion of CRELD2 are dramatically induced by ER stress. CRELD2 is ubiquitously expressed in multiple tissues at different levels, suggesting its crucial and diverse roles in different tissues. Recent studies suggest that CRELD2 is associated with cartilage/bone metabolism homeostasis and pathological conditions involving ER stress such as chronic liver diseases, cardiovascular diseases, kidney diseases, and cancer. Herein, we first summarize ER stress and then critically review recent advances in the knowledge of the characteristics and functions of CRELD2 in various human diseases. Furthermore, we highlight challenges and present future directions to elucidate the roles of CRELD2 in human health and disease.

## 1 Introduction

The endoplasmic reticulum (ER) is a multifunctional, dynamic intracellular organelle that plays an essential role in multiple physiological processes, including lipid metabolism; calcium homeostasis; and the synthesis, folding, and post-translational modifications of transmembrane and secreted proteins (1–3). The ER function is highly orchestrated by essential regulatory factors. However, various physiological and pathological stimuli can disrupt ER protein-folding capacity, thereby accumulating unfolded or misfolded proteins and disturbing ER homeostasis, which is referred to as ER stress (1–3). In response to ER stress, the unfolded protein response (UPR), which is considered as an integrated adaptive mechanism, is activated to restore protein homeostasis. However, persistent UPR activation can lead to a maladaptive cellular response, which has been implicated in various diseases including diabetes, obesity, fatty liver disease, cardiovascular diseases (CVDs), cancer, and kidney diseases (1, 3–13). Comprehensive understanding about the key factors involved in ER stress is crucial for elucidating the pathogenesis of these diseases and identifying novel therapeutic targets. Previous research has identified multiple novel proteins that play key roles during ER stress. Cysteine-rich epidermal growth factor (EGF)-like domain (CRELD) protein family member, CRELD2, was also recently identified as a putative ER stress-responsive protein (14–16).

CRELD protein family is highly conserved (17). *CRELD1*, the funding member of this family, is known as the *AVSD2* gene as mutations in *CRELD1* are associated with cardiac atrioventricular septal defects (AVSD) (17–26). CRELD2, another member of this family, is a factor that regulates the intracellular trafficking of acetylcholine receptor α4 and β2 subunits (27). Both CRELD1 and CRELD2 are multi-domain proteins, comprising varying numbers of EGF-like and calcium-binding EGF-like domains and a unique and highly conserved tryptophan-aspartic acid (WE) domain. The major structural difference between these two proteins is the presence of transmembrane domains in CRELD1 (absent in CRELD2) (15, 17, 28). This leads to distinct subcellular localizations and functions between CRELD members. Studies have revealed that CRELD1 is localized in the cytoplasm, probably mainly in the ER (29). Functional studies have demonstrated that CRELD1 is essential for heart development and immune cell function in mouse models (29–32). In contrast, CRELD2 is predominantly localized in the ER and Golgi apparatus and is spontaneously secreted (15). To date, evidence of the physiological and pathophysiological functions of CRELD2 is lacking. However, animal and cell models suggest that CRELD2 is a novel ER stress-responsive protein that might be implicated in ER homeostasis and ER stress-related diseases, including chronic liver diseases, CVDs, kidney diseases, and cancer. Herein, we summarize the characteristics and functions of CRELD2 based on current evidence and present future directions important for broadening our understanding of CRELD2 in health and disease.

## 2 ER stress and the UPR

The ER is an important organelle in eukaryotic cells that plays essential roles in orchestrating the homeostasis of at least one-third of the proteins within a cell and ensuring cell survival (2, 33). ER function is highly regulated by critical regulatory factors such as ER-resident enzymes and protein oxidoreductase chaperones (3). The impairment of these processes by both physiological and pathological stimuli, such as nutrient deprivation, aging, and hypoxia, may result in perturbed ER homeostasis, thereby accumulating misfolded or unfolded proteins in the ER lumen, a condition commonly known as ER stress (1–3). Following ER stress, UPR is triggered to either restore ER homeostasis or evoke cell death in cases of prolonged or unresolved ER stress. In response to mild to moderate ER stress, UPR is initiated to remove unfolded/misfolded proteins and restore ER homeostasis, resulting in “adaptive/cytoprotective” UPR. However, after severe or persistent ER stress, the UPR is hyperactivated, leading to “maladaptive/unchecked/terminal” UPR (3, 7, 12).

**Figure 1:**
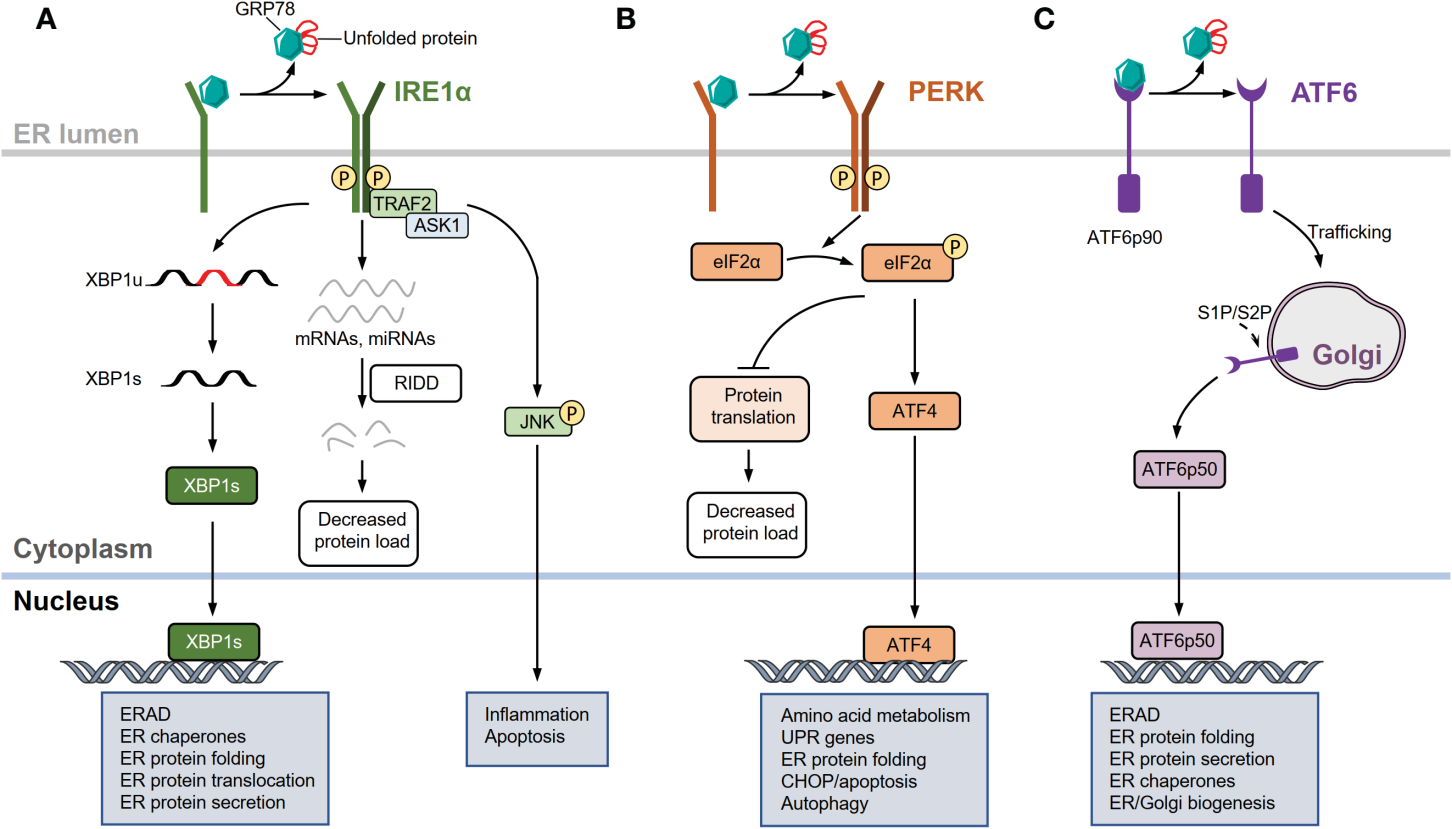
UPR signaling pathways. **(A)** Inositol-requiring enzyme 1 (IRE1α) signaling arm of the unfolded protein response (UPR). In response to endoplasmic reticulum (ER) stress, IRE1α is activated through dissociation from glucose- regulated protein 78 (GRP78), oligomerization, autophosphorylation, and subsequent allosteric activation of the cytosolic endonuclease domain. Activated IRE1α facilitates the unconventional splicing of *XBP1* (*XBP1u*) mRNA, resulting in active transcription factor XBP1s, which drives the expression of genes involved in restoring ER homeostasis. IRE1α also degrades select mRNAs and miRNAs through regulated IRE1-dependent decay (RIDD) to reduce protein load in the ER during intensive ER stress. In response to severe or unresolved ER stress, activated IRE1α recruits and binds to tumor necrosis factor receptor-associated factor 2 (TRAF2) and apoptosis signal-regulating kinase 1 (ASK1) to promote c-Jun N-terminal kinase (JNK) signaling, leading to the activation of apoptosis. **(B)** Protein kinase R (PKR)-like ER kinase (PERK) arm of the UPR. In response to ER stress, PERK is activated by dissociating from GRP78 and auto-phosphorylating after dimerization. Activated PERK selectively phosphorylates eukaryotic translation initiation factor 2 subunit-α (eIF2α), thereby attenuating protein translation. The mRNA of activating transcription factor 4 (ATF4) is preferentially translated following eIF2α phosphorylation, allowing it to upregulate genes involved in restoring ER homeostasis, amino acid metabolism, apoptosis, and autophagy. **(C)** Activating transcription factor 6 (ATF6) arm of the UPR. In response to ER stress, GRP78 dissociates from the luminal domain of full-length of ATF6 (ATF6p90), allowing ATF6 monomers to traffic to the cis-Golgi apparatus where they are proteolytically processed and cleaved by site-1 protease (S1P) and site-2 protease (S2P). This releases an active ATF6 transcription factor fragment (ATF6p50). The transcription factor localizes to the nucleus, inducing several genes involved in restoring ER homeostasis and ER/Golgi biogenesis.

In mammals, the UPR comprises three signaling pathways initiated by three ER transmembrane sensors: inositol-requiring enzyme 1 (IRE1α), protein kinase R (PKR)-like ER kinase (PERK), and activating transcription factor 6 (ATF6) (34–36). Under normal conditions, glucose-regulated protein 78 (GRP78), also known as heat shock protein family A member 5 or binding-immunoglobulin protein, which is a key ER-resident chaperone, binds to the luminal domains of all three UPR sensors to keep them inactive. However, during ER stress conditions, GRP78 preferentially binds to misfolded or unfolded proteins and is dissociated from these sensors, enabling their activation (3, 37) (**Figure 1**).

Among the UPR sensors, IRE1α is the most evolutionarily conserved arm and contains an ER stress-sensing luminal domain structure and a cytoplasmic effector domain with both RNA endonuclease (RNase) and kinase activities (37–41). Upon ER stress, IRE1α dissociates from GRP78 and is activated through several processes, including oligomerization, autophosphorylation, and subsequent allosteric activation of the cytosolic endonuclease domain (37–41). Activated IRE1α excises an inhibitory intron (an internal 26-nucleotide fragment) from cytosolic *XBP1* mRNA through its endonuclease activity, generating a frameshift when translating the spliced *XBP1* (*XBP1s*) isoform transcript (42–44). *XBP1s* encodes a functionally active basic leucine zipper (bZIP) transcription factor XBP1s, which regulates the transcription of multiple genes that are implicated in diverse biological pathways such as those that function in ER-associated protein degradation (ERAD) and folding proteins (37–39, 43). Additionally, the endonuclease activity of IRE1α can degrade a subset of mRNAs and miRNAs through regulated IRE1-dependent decay (RIDD), which reduces the abundance of proteins entering the ER during intensive ER stress (45–49). Moreover, activated IRE1α recruits and binds to tumor necrosis factor receptor-associated factor 2 (TRAF2) to promote c-Jun N-terminal kinase (JNK) signaling in response to severe or unresolved ER stimuli, thereby activating apoptotic programs (50, 51) (**Figure 1A**).

Similar to IRE1α, PERK is a type I transmembrane protein with a luminal ER stress-sensing domain and cytosolic Ser/Thr kinase domain (2, 33). Upon ER stress, PERK is activated through dissociation from GRP78 and subsequently dimerizes and auto-phosphorylates (37, 38). Activated PERK primarily acts as a kinase that phosphorylates the α subunit of eukaryotic initiation factor 2 (eIF2α), thereby attenuating protein translation and inducing activating transcription factor 4 (ATF4) (35, 37, 38, 52). Notably, PERK-mediated translational attenuation and transcriptional signaling are adaptive processes in response to acute ER stress, whereas chronic and prolonged PERK activation promotes apoptotic signaling through multiple mechanisms, including the induction of pro-apoptotic factors downstream of C/EBP homologous protein (CHOP) (33, 53) (**Figure 1B**).

Unlike IRE1α and PERK, ATF6 is a type II transmembrane protein with a cytosolic bZIP transcription factor dimerization domain that functions as a transcription factor rather than a kinase or endoribonuclease (1–3, 33). In response to acute ER stress, GRP78 dissociates from the luminal domain of the full-length ATF6 (ATF6p90). The increased population of ATF6 monomers traffics to the *cis*-Golgi apparatus, where they are proteolytically processed and cleaved by site-1 protease (S1P) and site-2 protease (S2P), releasing an active ATF6 transcription factor fragment (ATFp50) (54–56). This transcription factor localizes to the nucleus, inducing the expression of several genes involved in protein folding, lipid biogenesis, and ERAD (1, 33, 57–59) (**Figure 1C**).

Therefore, in response to ER stress, IRE1α, PERK, and ATF6 signaling pathways are collectively implicated in the coordination of the UPR though the following effects (1). First, these pathways attenuate translation and actively degrade RNA, reducing biosynthetic demands on the ER. Moreover, the protein refolding capacity can be enhanced to resolve misfolded/unfolded protein accumulation within the ER, and these pathways promote ubiquitination and proteasomal degradation *via* ERAD. Additionally, these pathways can mediate the delivery of misfolded/unfolded proteins from the ER to lysosomes for degradation *via* autophagy or to the cytosol for proteasomal processing. When these processes are dysregulated and not capable of restoring ER homeostasis, prolonged UPR activation and unresolved ER stress involve in cell apoptosis and death, which are associated with the pathogenesis of various diseases (1, 3–13, 60, 61).

## 3 Characteristics of CRELD2

### 3.1 Molecular structure of CRELD2

Fluorescence *in situ* hybridization showed that *CRELD2* maps to human chromosome 22q13 (28). Multiple splice variants are expressed by *CRELD2*, which encode for many transcripts that likely produce multiple protein isoforms (α, β, γ, δ, ϵ, ζ) (28). Sequence analysis revealed that each isoform has several overlapping motifs; however, they differ from each other in that they contain either tandem arrays of EGF- and/or calcium- binding EGF domains, or EGF/calcium-binding EGF domains and furin cysteine-rich domains (28). In contrast, they all have a WE-rich domain unique to the CRELD protein family, which is conserved across species (17, 28). CRELD2 is also highly conserved in orthologs and is identified in many vertebrates (28). There is also a murine *Creld2* ortholog with 69% identity to human *CRELD2* (17). Notably, the mouse *Creld2* gene presumably corresponds to the human *CRELD2α* gene (14).

**Figure 2:**
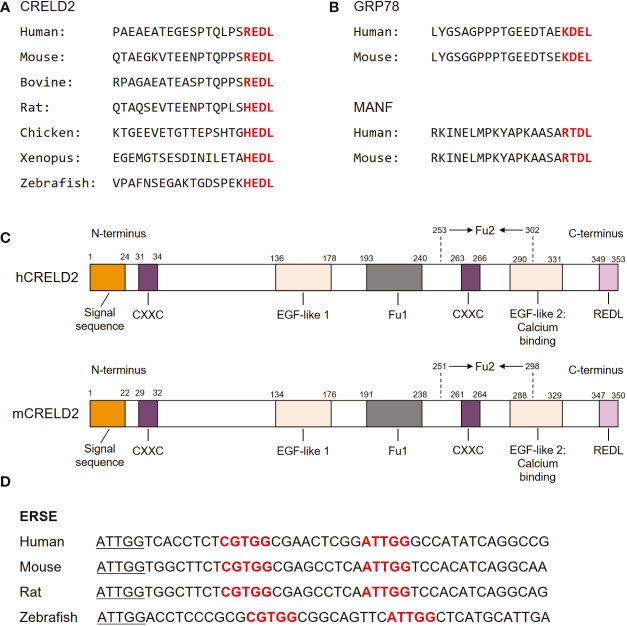
Molecular structure of CRELD2. **(A)** Comparison of amino acid sequences of CRELD2 at the C-terminus among various species. The conserved amino acids are shown in red font. **(B)** Four conserved C-terminal amino acids in GRP78 and mesencephalic astrocyte-derived neurotrophic factor (MANF) among species including human and mouse. The conserved amino acids are shown in red font. **(C)** Schematic of human and mouse CRELD2 protein. **(D)** Comparison of ERSE nucleotide sequences among various species. Conserved ERSE is shown in red font.

As the members of the *CRELD* family, *CRELD1* and *CRELD2* exhibit 51% homology in the nucleic acid sequences of the coding regions. At the amino acid level, they were 38% identical and 51% similar (17). Notably, CRELD1 and CRELD2 generally share similar domain structures, with a highly conserved WE region that has not been found in any non-homologous members, which is considered the hallmark feature of the CRELD protein family, followed by variations in EGF domains (28). In contrast to CRELD1, which has two type III transmembrane domains at the carboxyl(C)-terminus that anchors the protein to the cell surface, CRELD2 does not have any predicted transmembrane domains (15, 17, 28). The C-terminus of CRELD2 contains four (R/H)EDL amino acids, which are well conserved across species, including REDL for human, mouse, and bovine, and HEDL for rat, chicken, *Xenopus*, and zebrafish (15) (**Figure 2A**). These amino acids are reportedly similar to the C-terminal amino acids in several ER-resident proteins, such as KDEL for GRP78 (62, 63) and RTDL for mesencephalic astrocyte-derived neurotrophic factor (MANF) (64–67) (**Figure 2B**). In fact, many ER-resident proteins have a C-terminal KDEL (Lys-Asp-Glu-Leu) motif, which is the canonical sequence for retention of the ER chaperone including GRP78, while RTDL and (R/H)EDL are variants of KDEL sequence with similar functions (15, 66, 68). Studies have demonstrated that CRELD2 is primarily localized to the ER and Golgi apparatus and can be secreted into the extracellular space upon cellular stimuli, particularly ER stress (14, 15). The four amino acids domain in the C-terminus of CRELD2 is considered essential for the retention and secretion of CRELD2, which will be our focus in subsequent sections. Notably, studies have revealed that the amino(N)-terminus of CRELD2 is critical for its translocation into the ER-Golgi apparatus, whereas the deletion of its C-terminal region has no effect on its accumulation in the perinuclear region (15). Additionally, CRELD2 reportedly has numerous CXXC motifs (**Figure 2C**), and the N-terminal CXXC motif possesses protein isomerase activity (16). This pair of active protein disulfide isomerase (PDI)-like cysteine residues, CXXC, can shuttle between the disulfide and dithiol forms, thereby catalyzing thiol-disulfide oxidation, reduction, and isomerization, which are critical for correct disulfide bond formation and/or arrangement if incorrect bonds are formed (16, 69, 70). Therefore, its PDI-like activity further supports the idea that CRELD2 might participate in the quality control of proteins in the ER.

### 3.2 CRELD2 is an ER stress-inducible gene

The analysis of the upstream sequences of *CRELD2* revealed a functional promoter region embedded within a large CpG island (28). In 2009, Oh-hashi et al. performed a genomic sequence analysis of the promoter region of the mouse *Creld2* gene and identified a putative ER stress responsible element (ERSE; CGTGG-N9-ATTGG), which is highly conserved among different species (14) (**Figure 2D**). This conservation further indicates that CRELD2 may play a role in regulating cellular behavior during ER stress. They first identified that CRELD2 is predominantly localized in the ER and Golgi apparatus and is a novel ER stress-inducible gene (14). Specifically, in Neuro2a cells, *Creld2* mRNA was induced by treatment with the ER-inducing chemical agents, namely thapsigargin (Tg, a well-known inhibitor of Ca^2+^-ATPase), tunicamycin (Tm, an inhibitor of protein glycosylation), and brefeldin A (BFA, an inhibitor of ER-Golgi transport) (14). Furthermore, luciferase reporter analyses revealed that ATF6 overexpression drastically induced *Creld2* mRNA expression by directly binding to the ERSE of the *Creld2* gene. Mutations in the mouse *Creld2* promoter ERSE considerably decreased both basal activity and responsiveness toward ER stress stimuli (14).

Additionally, further studies confirmed that CRELD2 is responsive to ER stress stimuli. For example, neonatal rat cardiomyocytes treated with Tm significantly upregulated *Creld2*, as well as several other genes involved in ER stress. This upregulation was significantly attenuated by salubrinal and its derivatives, which are the inhibitors of ER stress (71). Additionally, mouse podocytes treated with either Tm or Tg for 24 h remarkably induced CRELD2 expression (72), indicating that the upregulation of CRELD2 induced by ER stress stimuli is a general and not cell-type-specific response. Moreover, *in vivo* studies also found that CRELD2 protein expression in the mouse liver was considerably induced 24 h after intraperitoneal injection of Tm (73). These results suggest that CRELD2 may be involved in the processing of proteins and may be a novel mediator in regulating the onset and progression of various ER stress-associated diseases.

### 3.3 ER stress induces CRELD2 secretion

**Figure 3:**
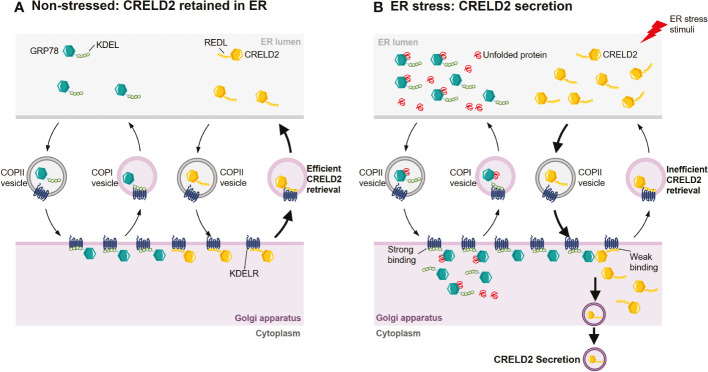
Hypothetical mechanism for the ER stress-induced CRELD2 secretion. Although ER-resident chaperones including GRP78 appear to be located solely in the ER lumen, they are widely distributed throughout the cell owing to a dynamic equilibrium achieved through retention and bidirectional transport between organelles. **(A)** Under normal conditions, GRP78 and CRELD2 can be trafficked to Golgi apparatus from ER *via* coat protein complexes II (COPII) vesicles. In the Golgi apparatus, the KDEL sequence of GRP78 and REDL sequence of CRELD2 can be recognized by the KDEL receptor (KDELR), which is located in the *cis*-Golgi. The binding of GRP78 or CRELD2 to KDELR triggers the incorporation of receptor-protein complex into vesicles such as COPI, thereby efficiently transporting them back to the ER. **(B)** Upon ER stress, the levels of several ER-inducible proteins, including GRP78 and CRELD2, are upregulated; however, the relative expression of KDELR is not increased. Thus, GRP78 containing perfect ER retention motifs with a high affinity for KDELR will compete for KDELR with CRELD2 containing a low affinity motif. Therefore, CRELD2 that escapes to the Golgi apparatus cannot be efficiently retrieved back to the ER, allowing CRELD2 to be secreted.

CRELD2 is both an ER-resident protein and a secretory factor (14, 15). However, the mechanisms that regulate the intracellular transportation and secretion of CRELD2 are poorly understood. In 2011, Oh-hashi et al. found that the N-terminus of CRELD2 is necessary for its translocation into the ER-Golgi apparatus (15). Additionally, the disruption of the Golgi apparatus by BFA almost completely prevents CRELD2 secretion (15, 74), suggesting that CRELD2 might be secreted *via* the ER-Golgi apparatus. Moreover, the overexpression of dominant negative mutant *Sar1*, a component of cytoplasmic vesicle coat protein complex II (COPII), drastically suppressed the spontaneous secretion of CRELD2. Sar1 plays an essential role in early step in vesicle budding and regulating vesicular transport from the ER to the Golgi (75, 76), and this further supports that COPII-mediated transport from the ER to the Golgi apparatus is the main pathway for CRELD2 trafficking (15) (**Figure 3A**). Despite this evidence, further studies are warranted to comprehensively understand the mechanism that regulates cellular transport of CRELD2.

The conditions under which CRELD2 secretion is induced are still not comprehensively elucidated. However, both *in vitro* and *in vivo* studies have demonstrated that CRELD2 secretion is drastically induced upon ER stress. For example, cells subjected to ER stress by chemical agents such as Tm, Tg, and BFA, or by culturing cells in serum-free medium showed increased CRELD2 secretion (15, 73). In ER stress-related disease models, such as matrilin-3 *(Matn3)* and collagen type X alpha 1 chain *(Col10a1)* mutant growth plates, CRELD2 was also detectable at significant levels in the extracellular matrix (ECM) but not in wild-type controls (16). Additionally, CRELD2 is rapidly secreted when renal cells are subjected to ER stress in mouse models of podocyte ER stress-induced nephrotic syndrome (NS) and Tm- or I/R-induced acute kidney injury (AKI) (72). The observation that CRELD2 is an ER-resident protein and can be secreted under induced ER stress suggests that it might act as a multifunctional factor both in the ER-Golgi apparatus as well as in the extracellular space under normal and pathophysiological conditions.

Studies have revealed that the four-amino acid sequence (R/H)EDL in the C-terminal region of CRELD2 regulates its secretion. For example, in HEK293 cells, the modification of the C-terminal region, such as the deletion of four C-terminal amino acids from mouse CRELD2 or addition of tag-peptides to its C-terminus, drastically enhanced CRELD2 secretion (15). Additionally, the overexpression of wild-type GRP78, but not mutant GRP78 lacking the C-terminal KDEL sequence, remarkably enhanced CRELD2 secretion, whereas mutant CRELD2 at the C-terminus did not show any upregulation (15). Therefore, CRELD2 is a novel secretory factor regulated by GRP78, whereas the comprehensive molecular mechanism remains unresolved. However, the KDEL receptor (KDELR) competition hypothesis was also proposed (15, 16) (**Figures 3A, B**). The four highly conserved amino acids in CRELD2 are similar to the C-terminal amino acids in GRP78, which also possesses the well-known ER-resident signal sequence KDEL (62, 63). The secretion of many ER-resident proteins is restricted *via* this ER retention motif, facilitating retrieval transport from the Golgi apparatus *via* binding to KDELRs, which are located in the intermediate compartment or in the *cis*-Golgi apparatus, with high affinity (68, 77–84). Similarly, the putative ER retention motif REDL at the C-terminus enables CRELD2 to be retained in the ER (15). Under normal conditions, both REDL and KDEL trafficked to Golgi apparatus can bind with KDELRs, thereby efficiently transporting them back to the ER. However, during ER stress, the relative levels of multiple ER stress-inducible proteins such as GRP78 and CRELD2 are upregulated, whereas the relative expression of KDELRs are not increased (16, 85). Thus, GRP78, containing perfect ER retention motifs that have high affinity for the KDELRs, would compete with CRELD2, containing a motif that has a low binding affinity, thereby allowing CRELD2 to escape the ER and ultimately be secreted (15, 16) (**Figures 3A, B**).

Further studies have revealed that, additionally to GRP78, MANF can enhance CRELD2 secretion (86). Similar to CRELD2, MANF has recently been identified as an ER stress-responsive protein (65–67, 85, 87–92). MANF possesses ER-resident motifs at its proximal C-terminus, RTDL, which regulates MANF secretion upon ER stress (64–67). Studies have revealed that in both HEK293 and COS7 cell lines, co-transfection with CRELD2 and MANF remarkably increased the secretion of CRELD2 into the extracellular space (86). This effect highly depends on both of the four C-terminal amino acid sequences (RTDL in MANF and REDL in CRELD2) because the deletion of these four C-terminal amino acids terminated the increased secretion of CRELD2 induced by the co-expression of MANF (86). Notably, the data showed that the increase in CRELD2 secretion by MANF overexpression might not be simply due to the expression of intrinsic ER-resident chaperones or a result of competition between the two proteins for KDELRs (86). Further studies have revealed that the overexpression of mouse cerebral dopamine neurotrophic factor (CDNF), a paralogous protein of MANF that also contains the KDEL-like motif of the ER retrieval signal at its C-terminus, hardly affected the co-transfected CRELD2 secretion (93). Moreover, the inhibition of vacuolar ATPase, a proton pump that regulates the pH of several intracellular compartments, remarkably induced CRELD2 secretion without relying on its C-terminal structure (74). These results provide valuable insights into the mechanism of CRELD2 secretion regulation, which warrants further investigation.

### 3.4 Tissue expression of CRELD2

Several studies have confirmed the ubiquitous expression of *CRELD2*. In 2005, through northern blotting, Ortiz et al. first revealed that the high expression levels of *CRELD2* were observed in the skeletal muscle, heart, liver, kidney, and placenta of human tissues (28). In 2006, Maslen et al. conducted intensive studies in different human tissues by combining both northern blot and RNA dot blot (28). They demonstrated that *CRELD2* was ubiquitously expressed during development and in mature tissues, although a broad range of signal intensities were observed. Additionally, the most prominent signals of *CRELD2* in adult tissues occur in pancreas, stomach, duodenum, salivary gland, thyroid gland, appendix, and trachea, which generally overlap with the expression pattern for *CRELD1* (17, 28). However, in fetal tissues, *CRELD2* is highly expressed in the lung, liver, thymus, spleen, and heart (28).

Additional to its expression pattern in humans, the ubiquitous expression of CRELD2 has been confirmed in rodents (73, 94). In 2018, Oh-hashi et al. evaluated the mRNA and protein expression patterns of CRELD2 in different adult mouse tissues (73). They found that the *Creld2* mRNA was present in almost all tissues tested, including the brain, heart, lung, liver, spleen, kidney, stomach, and small intestine, but not in skeletal muscles. However, CRELD2 protein expression in the heart and skeletal muscle was negligible, and that in the brain was relatively low (73). To understand the discrepancy between CRELD2 mRNA and protein expression, they further examined the stability of CRELD2 protein in Neuro2a cells through treatment with the protein synthesis inhibitor cycloheximide and/or the proteasome inhibitor MG132 together with the ER stress agent Tg. However, the protein levels of CRELD2 were hardly affected by each treatment (73). Until now, of the reason for this discrepancy remains unclear, which may be important for us to further understand the characteristics and functions of CRELD2.

## 4 CRELD2 in disease models

As previously discussed, CRELD2 is a novel ER stress-responsive protein, and its expression and secretion can be drastically enhanced by ER stress. In this section, we provide a comprehensive overview of the recent advances in the knowledge about CRELD2 in various disease models (**Table 1**).

**Table 1:** Summary of current studies associating CRELD2 with diseases.

Diseases or pathology	Samples or models	Main findings	Refs
NAFLD	NAFL patients *vs.* control	- No difference was observed in CRELD2 expression in the liver.	(94)
	NASH *vs.* NAFL patients	- CRELD2 expression in the liver was upregulated in male NASH patients, but not in that of female patients; - Serum CRELD2 concentration was inversely correlated with SAF scores in male patients, whereas no correlation was observed in female patients.	(94)
	Mice i.p. injected with Tm for 24 h	- CRELD2 expression in the liver was upregulated.	(73)
	*Creld2* ^-/-^ *vs.* control mice fed with a chow diet	- *Creld2* ^-/-^ mice gained less weight.	(94)
	*Creld2* ^-/-^ *vs.* control mice fed with a high-fat diet	- *Creld2* ^-/-^ mice gained less weight; - *Creld2* ^-/-^ mice developed insulin resistance; - *Creld2* ^-/-^ livers contained fewer lipids; - *Creld2* ^-/-^ livers exhibited more apoptosis - *Creld2* ^-/-^ livers showed similar UPR activation.	(94)
	*Creld2* ^-/-^ *vs.* control mice i.p. injected with Tm for 48 h	- *Creld2* ^-/-^ livers contained more lipids; - *Creld2* ^-/-^ livers showed exacerbated ER stress.	(94)
	*Creld2* ^-/-^ *vs.* control mice at ~1 year-old	- *Creld2* ^-/-^ livers accumulated more lipids in male mice, but not in that of female mice.	(94)
Alcoholic liver disease	*Grp78* ^-/-^ mice fed with long-term alcohol	- In the older *Grp78* ^-/-^ females, stronger effects of the alcohol on methylation of CpG islands at promoter regions of *Creld2* accompanied with other genes involved in the ERAD.	(95)
Cardiovascular diseases	Human aneurysmatic samples *vs.* healthy aortas	- *Creld2* mRNA expression upregulated.	(96)
	VSMC from control donors or MFS patients	- A *FBN1* 3’UTR mutation was identified in VSMC from MFS patients; - In the non-dilated aortic zone of MFS patients with 3’UTR mutation, *CRELD2* accompanied with other UPR genes upregulated	(97)
	Primary neonatal rat cardiomyocytes treated with Tm	- *Creld2* accompanied with other UPR genes expression upregulated; - Salubrinal, an ER stress inhibitor, reversed the upregulation of *Creld2* induced by Tm.	(71)
Cartilage and bone metabolism	A *matn3* mutant mouse model of MED	- *Creld2* was the most highly upregulated gene in the chondrocytes.	(98)
	Schmid and Cog mouse models with ER stress-related growth plate disease	- CRELD2 expression in the hypertrophic zones was significantly upregulated.	(99)
	Mouse femoral heads cartilage cultured with or without IL-1α	- *Creld2* accompanied with other ER stress-responsive genes were upregulated in cartilage upon IL-1α treatment.	(100)
	Cell lines and mouse models of chondrodysplasias	- CRELD2 protein levels were upregulated in chondrodysplasias caused by mutations in *Matn3* or *Col10a1*, but not *Comp*; - CRELD2 secreted into the ECM of ER stress-induced by *matn3* or *Col10a1* mutation, but not *Comp*; - CRELD2 interacted with substrates including matrilin-3, but not with COMP.	(16)
	MSCs stimulated with BMP9	- *Creld2* was among the top up-regulated genes; - SMAD1/5/8 directly bound to the *Creld2* promoter; - *Creld2* overexpression promoted BMP9-induced bone formation and matrix mineralization; - Silencing *Creld2* expression suppressed BMP9-induced osteogenic differentiation.	(101)
	MSCs transduced with BMP9 and *Creld2*, *simCreld2*, or Control s.c. injected into the flanks of athymic nude mice	- Exogenous *Creld2* enhanced BMP9-induced ectopic bone formation; - Silencing *Creld2* inhibited BMP9-induced ectopic bone formation.	(101)
	Cartilage- or bone- specific *Creld2* KO mouse models	- Cartilage-specific *Creld2* KO mice displayed a chondrodysplasia-like phenotype; - Bone-specific *Creld2* KO mice exhibited an osteopenic phenotype; - CRELD2 acted as a novel chaperone for LRP1, promoting its transport to the cell surface, subsequently modulating WNT signaling during the differentiation and maturation of chondrocytes and osteoblasts.	(102)
	RAW264.7 cell line or mouse primary osteoclast stimulated with RANKL	- CRELD2 expression downregulated following osteoclastogenic differentiation; - *Creld2* overexpression impaired osteoclast differentiation; -Mechanistically, CRELD2 blocked calcium release from the ER, reduced calcium-dependent calcineurin activity and the subsequent nuclear translocation of NFATc1.	(103)
Cancer	MB114 cell line	-CRELD2 expression increased cell invasion, and promoted a trend toward enhanced angiogenic sprouting.	(104)
	Cell models and clinical tumor samples of prostate cancer	-CRELD2 was characterized to be one of novel androgen receptor targets.	(105)
	Human RCC cell lines	- *Creld2* was one of the target genes of miR-451a; - High *Creld2* expression was associated with poor prognosis in patients with RCC.	(106)
	HCC *vs.* non-tumor tissues from databases	- CRELD2 expression was significantly upregulated in HCC tissues; - CRELD2 was found to be an adverse prognostic biomarker for disease-free survival in HCC.	(107)
	Cell and mouse models of breast cancer	-ROCK activation in mammary cancers yielded a tumour-promoting fibroblast phenotype through paracrine CRELD2; - ROCK activation in mammary cancers induced ATF4-regulated *Creld2* transcription.	(108)
	Normal human breast and invasive breast carcinoma samples	- CRELD2 expression upregulated in samples of invasive breast carcinoma; - High CRELD2 expression is associated with progressive human breast cancer.	(108)
Prosthetic joint infection	Synovial fluid from patients required hip/knee revision surgery	- CRELD2, as well as IL-16 and IL-18, would be potential biomarkers for prosthetic joint infection diagnosis.	(109)
ER stress-related kidney diseases	Primary mouse podocytes and podocyte ER stress-induced NS mouse model	- CRELD2 cellular secretion and urinary excretion coincide with podocyte ER stress during the development of proteinuria and can be detected at the early stage of the disease.	(72)
	AKI mouse model induced by Tm	- Tubular cell ER stress increased urinary CRELD2 excretion prior to the decline in kidney function.	(72)
	AKI mouse model induced by I/R	-Urinary CRELD2 excretion can serve as a mechanistic biomarker for ER-stressed tubular cells in the early phase of I/R-induced AKI.	(72)
	Mouse with bilateral renal pedicle clamping	- CRELD2 excretion levels correlated with the duration of renal ischemia.	(72)
	Pediatric patients received congenital cardiac surgery	-Urinary CRELD2 elevation within postoperative 6 hours was significantly associated with higher risk or severe AKI and other adverse clinical outcomes after pediatric cardiac surgery.	(72)
	ADTKD-*UMOD* patients *vs.* controls	-Urinary CRELD2 levels were markedly increased in patients with ADTKD caused by mutations in *UMOD*.	(72)
	Human fetal and postnatal human kidney samples	-Immunohistochemical study found a high expression of CRELD2 in kidney structures during normal human fetal and postnatal kidney development.	(110)

ADTKD-UMOD, autosomal dominant tubulointerstitial kidney disease due to mutations in the UMOD gene; AKI, acute kidney injury; ATF4, activating transcription factor 4; BMP9, bone morphogenetic protein 9; ECM, extracellular matrix; ER, endoplasmic reticulum; ERAD, ER-associated protein degradation; HCC, hepatocellular carcinoma; i.p., intraperitoneal; I/R, ischemia-reperfusion; KO, knockout; LRP1, lipoprotein receptor-related protein 1; MED, multiple epiphyseal dysplasia; MFS, Marfan syndrome; MSCs, mesenchymal stem cells; NAFL, non-alcoholic fatty liver; NAFLD, non-alcoholic fatty liver disease; NASH, non-alcoholic steatohepatitis; NFATc1, nuclear factor of activated T cells 1; NS, nephrotic syndrome; RANKL, receptor activator of nuclear factor kappa-B ligand; RCC, renal cell carcinoma; ROCK, Rho-associated kinase; SAF, steatosis, activity and fibrosis score; s.c., subcutaneous; Tm, tunicamycin; UPR, unfolded protein response; VSMC, vascular smooth muscle cells.

### 4.1 CRELD2 in chronic liver diseases

ER stress reportedly contributes to the onset and progression of multiple liver diseases including non-alcoholic fatty liver disease (NAFLD) and alcoholic liver diseases (3, 111–116). Although CRELD2 is involved in liver metabolism homeostasis, its exact role and molecular mechanisms remain largely undefined. The involvement of CRELD2 in liver metabolism was first reported by Nohara et al. Their results showed that gestational arsenic exposure significantly increased *Creld2* expression in the liver of adult male offspring mice, accompanied by an increase in intracellular triglyceride accumulation in the liver (117). Subsequent studies further found that intraperitoneal administration of Tm for 24 h drastically induced CRELD2 protein expression in mouse livers (73). Moreover, in older *Grp78* ^-/-^ mice, increased effects of alcohol on the methylation of CpG islands in the promoter regions of genes involved in ERAD, including *Creld2*, were detected (95). However, a direct causal link between CRELD2 and liver disease has not been demonstrated in these studies.

Through intensive studies on mouse models and human samples, Kern et al. recently revealed that CRELD2 plays a critical role in liver homeostasis (94). They generated a *Creld2* knockout (*Creld2* ^-/-^) mouse model to investigate CRELD2 function *in vivo* (94). Their results showed that *Creld2* ^-/-^ mice exhibited a reduction in body weight under both a chow diet and high-fat diet (HFD) compared to their controls but developed insulin resistance. Additionally, *Creld2* ^-/-^ livers generally contained fewer lipids, which could account for lower body weight. Notably, their results clearly showed that 12 weeks of HFD did not induce ER stress in the liver, and neither transcriptome nor protein expression analysis revealed a lipid-driven ER stress response in the livers of *Creld2* ^-/-^ mice. Moreover, this study revealed that the adipose weights of *Creld2* ^-/-^ mice were significantly reduced (94). Another study also identified several genes, including *Creld2*, that were associated with feed efficiency using RNA sequencing in rumen papillae from steers (118). All these results suggest that CRELD2 plays a role in regulating whole-body energy balance, which remains elusive and warrants further investigation. To test whether CRELD2 is involved in the resolution of ER stress in the liver, *Creld2* ^-/-^ mice and littermate controls were intraperitoneally injected with Tm or vehicle. Inducing ER stress *via* Tm administration exacerbated ER stress in *Creld2* ^-/-^ mice, as indicated by upregulated GRP78 expression, and consequently increased hepatic lipid storage. Mechanistic studies have revealed that CRELD2 interacts with several chaperones and enzymes whose activity is required to overcome cellular stress, including GRP78, thioredoxin domain-containing 5, and glutathione S-transferase Mu2 (94, 119). These data further support the hypothesis that CRELD2 is involved in ER stress and the UPR, and CRELD2 function is required to maintain hepatic homeostasis under ER stress responses in mice.

Additionally, Kern et al. investigated whether human CRELD2 plays a role in the pathophysiological conditions of the liver. They found sex dimorphism in the expression levels of CRELD2 in the human liver, where both the mRNA and protein levels of CRELD2 were significantly upregulated in male patients with non-alcoholic steatohepatitis (NASH); however, expression in female patients remained unaffected (94). Notably, control patients showed expression similar to that of patients with simple steatosis. Furthermore, they found an inverse correlation between serum concentrations of CRELD2 and Steatosis, Activity, and Fibrosis (SAF) scores in male patients (94). These results indicate that CRELD2 exhibits a sex-specific function during NAFLD in humans, and upregulated CRELD2 expression in the liver is likely associated with a decrease in serum CRELD2 concentration, which is presumably associated with progression to NASH in male patients. Similarly, a sex dimorphism was also observed in aged mice, with only *Creld2* ^-/-^ males developing steatosis (94). Based on the results obtained from both mouse models and human samples, CRELD2 likely plays an essential role in liver metabolic homeostasis; however, further studies are warranted to validate this.

### 4.2 CRELD2 in CVDs

ER stress reportedly involves in the pathogenesis of CVDs, including hypertension, atherosclerosis, heart failure, and myocardial atrophy (6, 12). In primary neonatal rat cardiomyocytes, Tm treatment significantly induced the mRNA expression of *Creld2*, accompanied by other UPR genes, and this upregulation was remarkably reversed by treatment with salubrinal, which is an ER stress inhibitor (71). Further studies revealed high expression of CRELD2 and other ER stress-related markers in human aneurysmal samples (96). This is accompanied by exacerbated apoptosis, high reactive oxygen species production, and a reduction in mitochondrial biogenesis in the vascular wall of abdominal aortic aneurysm, a degenerative vascular disease with a complex etiology (96). Marfan syndrome (MFS) is associated with mutations in the protein fibrillin-1 (FBN1), which affects the integrity of connective tissue elastic fibers. The most severe clinical outcome of MFS is cardiovascular system abnormalities characterized by progressive aortic root enlargement and ascending aortic aneurysm (97, 120, 121). Siegert et al. found a nearly undescribed *FBN1* 3’UTR mutation accompanied by a well-defined gene ontological ER stress response in the non-dilated aortic zone, which was confirmed by the increased expression of MANF, GRP78, and CRELD2 (97). These results indicate that CRELD2 is associated with CVD pathogenesis. However, comprehensively elucidating the roles and mechanisms of CRELD2 in CVDs using various animal and cell models is necessary.

### 4.3 CRELD2 in cartilage and bone metabolism

ER stress is also implicated in the disturbance of cartilage and bone metabolism (60, 61, 122, 123). *In vitro* and *in vivo* studies demonstrate that CRELD2 plays an important role in these processes. The association between CRELD2 and cartilage metabolism was first reported by Rajpar et al. (98). They revealed that *Creld2* was the most highly upregulated gene in chondrocytes from a *Matn3* mutant model of multiple epiphyseal dysplasia (MED), which is a clinically variable and genetically heterogeneous chondrodysplasia characterized by mild short stature, joint pain, and stiffness and early onset osteoarthritis (98). Further studies have also revealed that CRELD2 expression in the hypertrophic zones of ER stress-related growth plate disease mouse models (Schmid and Cog) is significantly upregulated (99). Notably, MED, Schmid, and Cog are characterized by prolonged ER stress. These data also revealed that the protein levels of CRELD2 were upregulated in cell and mouse models of chondrodysplasia caused by mutations in *Matn3* or *Col10a1*, but not in cartilage oligomeric matrix protein *(Comp)* (16). Notably, upon ER stress induced by *matn3* mutation *in vitro* and *in vivo*, CRELD2 was also secreted into the ECM (16). Moreover, substrate-trapping experiments confirmed that PDI-like activity enabled CRELD2 to interact with several substrates, including MATN3, laminin 5 β3, type VI collagen, and thrombospondin 1 (16). Additionally, new elements of ER stress in IL-1α-treated cartilage reportedly involve *Creld2* (100), further supporting that CRELD2 might be associated with cartilage homeostasis.

Additionally, CRELD2 participates in bone development and homeostasis. For example, *Creld2* is among the top upregulated genes in bone morphogenetic protein 9 (BMP9)-stimulated osteogenic differentiation of mesenchymal stem cells (101). ChIP assays revealed that SMAD1/5/8 directly binds to the *Creld2* promoter in a BMP9-dependent manner. Moreover, both *in vitro* and *in vivo* studies have revealed that exogenous *Creld2* promotes BMP9-induced bone formation and matrix mineralization; conversely, silencing *Creld2* expression suppresses BMP9-induced osteogenic differentiation (101). These results confirmed that CRELD2 plays an important role in BMP9-induced terminal osteogenic differentiation, although the specific mechanism still needs to be thoroughly investigated (101). Dennis et al. recently found that mice with cartilage-specific deletion of *Creld2* had a chondrodysplasia-like phenotype with abnormal cartilage growth plates; however, the deletion of *Creld2* in bone results in osteopenia, with a low bone density and altered trabecular architecture (102). Mechanistically, CRELD2, which possesses PDI-like activity, acts as a novel chaperone for LRP1, promoting its transport to the cell surface and subsequently modulating WNT signaling during the differentiation and maturation of chondrocytes and osteoblasts (102). They recently further revealed the critical role of CRELD2 in osteoclast differentiation (103). CRELD2 expression was significantly downregulated following osteoclastogenic differentiation in both the RAW264.7 cell line and mouse primary osteoclast precursors, and the overexpression of *Creld2* impaired osteoclast differentiation *in vitro*. Mechanistic analysis revealed that CRELD2 expression blocks calcium release from the ER. This impairs osteoclast differentiation due to reduced calcium-dependent calcineurin activity and nuclear translocation of nuclear factor of activated T cells 1 (NFATc1), a master regulator of osteoclastogenesis (103). Interestingly, another study has shown that the first member of the CRELD family of proteins, CRELD1, promotes NFATc1 dephosphorylation and its subsequent nuclear translocation by directly binding to and modulating calcineurin activity (29, 31). The opposite effect on NFATc1 translocation mediated by CRELD1 and CRELD2 indicates that these two proteins fulfill distinct cellular functions, possibly due to their structural differences. As for CRELD2, conducting *in vivo* studies in animal models and clinical studies in humans is necessary, as these comprehensive studies could broaden our understanding of the critical role of CRELD2 in skeletal and other human diseases.

### 4.4 CRELD2 in cancer

Increasing evidence has established that ER stress and UPR pathways play essential roles in tumor progression and survival (1, 6, 124). As a novel ER stress-responsive gene, *Creld2* is highly correlated with cancer development, although the detailed underlying pathophysiology remains unclear. For example, *in vitro* studies revealed that CRELD2 expression significantly increased MB114 cell invasion and promoted a trend toward enhanced angiogenic sprouting, thereby suggesting that CRELD2 might be a mediator of tumor angiogenesis (104). Additionally, CRELD2 was reported as a novel androgen receptor target in prostate cancer (105). Yamada et al. identified 16 putative oncogenic targets of miR-451a regulation in renal cell carcinoma. Among these, *CRELD2* was one of the target genes whose expression was significantly associated with poor prognosis in patients with renal cell carcinoma by TCGA database analyses (106). Additionally, CRELD2 gene and protein expressions are significantly upregulated in hepatocellular carcinoma (HCC) than in non-tumor tissues, and CRELD2 is associated with poor overall survival and disease-free survival in HCC (107). However, the specific role of CRELD2 in carcinogenesis remains unelucidated.

Sarah et al. recently established a causal link between CRELD2 and cancer (108). Their results showed that CRELD2 is a paracrine factor that underlines PERK-mediated cancer-associated fibroblast education downstream of Rho-associated kinase (ROCK) *via* an analysis of tumors from patients and mice (108). They found that *Creld2* is regulated by PERK-modulated ATF4 *via* a putative C/ebp-Atf response element (CARE) in the promoter sequences of *Creld2*, and CRELD2 depletion suppresses tumor progression, indicating that the paracrine ROCK-PERK-ATF4-CRELD2 axis promotes breast cancer progression. They also revealed that high CRELD2 expression was associated with progressive human breast cancer (108). Therefore, these results establish that CRELD2 is a driver of tumor progression, and targeting CRELD2 may be a suitable anticancer therapy.

### 4.5 CRELD2 secretion as disease biomarker

The observation that CRELD2 is secreted under increased ER stress suggests that it may be exploited as a soluble extracellular biomarker of ER stress-related diseases. Emerging evidence has characterized CRELD2 as a biomarker in human body fluids for molecular phenotyping of pathogenetic processes. For example, synovial fluid CRELD2 has been identified as a potential biomarker for prosthetic joint infection (109). Moreover, male NASH patients showed an inverse correlation between serum CRELD2 concentration and NASH progression, with low CRELD2 levels at high SAF scores (94).

Moreover, CRELD2 is characterized as an early, sensitive, non-invasive, and mechanistic biomarker in the urine in various ER stress-mediated kidney diseases, including hereditary NS, ischemic AKI, and autosomal dominant tubulointerstitial kidney disease (ADTKD), due to mutations in the *UMOD* gene encoding uromodulin (72, 125–127). In a podocyte ER stress-induced NS mouse model, CRELD2 cellular secretion and urinary excretion coincided with podocyte ER stress during the development of proteinuria and could be detected at an early stage of the disease. Moreover, tubular cell ER stress increases urinary CRELD2 excretion prior to subsequent decline in kidney function or histologic changes due to ER stress-induced AKI. Therefore, urinary CRELD2 excretion can serve as a mechanistic biomarker for ER-stressed tubular cells in the early phase of I/R-induced AKI (72). Additionally, these data have shown that early postoperative urinary CRELD2 elevation is significantly associated with severe AKI and other adverse outcomes following pediatric cardiac surgery. Urinary CRELD2 can be used to distinguish between controls and patients with early kidney disease (72). These data suggest that CRELD2 may be a promising ER stress biomarker with potential application in early diagnosis, risk stratification, treatment response monitoring, and guiding the development of ER stress modulators in targeted patient groups. Nevertheless, further clinical studies in a multi-institutional setting with a larger patient cohort are warranted. Notably, a recent immunohistochemical study on human fetal and postnatal human kidney samples revealed a high expression of CRELD2 in kidney structures during normal human fetal and postnatal kidney development (110). This implies that CRELD2 may play an essential role in kidney homeostasis, which also warrants further investigation.

## 5 Concluding remarks and future directions

Over the past decades, increasing evidence has revealed the characteristics and functions of CRELD2. Molecular studies have demonstrated that CRELD2 acts as both an ER-resident protein and a secretory factor. Various cellular stimuli, particularly ER stress, remarkably enhance CRELD2 expression and secretion, as demonstrated by multiple *in vitro* and *in vivo* studies. Expression pattern analysis revealed that CRELD2 is ubiquitously expressed in multiple tissues to different extents, suggesting that CRELD2 may play important but diverse roles in different organs, tissues, and in different cell types. Moreover, CRELD2 is associated with various diseases and has crucial roles in various physiological and pathological processes, including liver metabolism homeostasis, cartilage and bone metabolism, and cancer progression and survival, as well as potential role as a biomarker for kidney diseases. However, despite significant progress, many outstanding concerns regarding CRELD2 remain unaddressed.

First, multiple isoforms of human CRELD2 protein cause variations in their subcellular localization, expression patterns, and functions, which have not yet been comprehensively elucidated. In addition, although there is evidence regarding the mechanism through which ER stress induces CRELD2 expression, it remains unclear whether other mechanisms are also involved in this process. Additional to the upregulation of CRELD2 expression, ER stress can drastically enhance its secretion. However, information on the changes in circulating CRELD2 levels in ER stress-related diseases, including diabetes, obesity, inflammation, NAFLD, CVDs, kidney diseases, and cancer, is still lacking. The source of circulating CRELD2 in these pathological conditions also remains unknown. Moreover, MANF overexpression significantly increased CRELD2 secretion. However, the exact mechanism underlying this process remains unclear. Whether CRELD2 plays a role in the function of MANF and whether there exists any link between these two proteins also remain unknown and require further studies. Similar to MANF, CRELD2 may possess a dual-function model (intracellular and extracellular functions). However, knowledge about its functions and mechanisms, particularly that of extracellular CRELD2, is still lacking. Current evidence has revealed an association between CRELD2 and various human diseases, whereas the direct causal link between CRELD2 and the pathogenesis of diseases remains poorly understood. Further investigations may provide clues as to whether the addition of exogenous CRELD2 or blocking endogenous CRELD2 is therapeutically beneficial for human diseases. This should also be validated in mouse models using CRELD2 peptides or specific antibodies. Addressing these gaps in knowledge would be beneficial for uncovering the pathophysiological significance of CRELD2 and its potential clinical applications.

## Author contributions

JH contributed to the guidance of this review and gave the final approval of the version to be published. QT drafted the manuscript. QL, YL, and LM prepared the figures. All authors read and approved the final manuscript.
